# Commentary: Efficacy and safety of HSK21542 for pruritus management in hemodialysis patients: a multicenter, randomized, double-blind, placebo-controlled trial

**DOI:** 10.3389/fphar.2025.1689698

**Published:** 2025-10-17

**Authors:** Steven Fishbane

**Affiliations:** Division of Nephrology, Zucker School of Medicine at Hofstra/Northwell, Great Neck, NY, United States

**Keywords:** pruritus, kidney, hemodialysis, clincial trials, HSK21542

## Introduction

I read with interest the article by Pan et al. (2025) on HSK21542 (Anrikefon) for managing CKD-associated pruritus (CKD-aP) in haemodialysis patients. This Phase II trial addresses an important unmet need for patients that suffer from CKD-aP. However, I have identified material errors in the authors’ interpretation of their results, as well as some uncontextualized comparisons. Here I highlight these, in the interest of scientific dialogue.

## Efficacy and safety claims

The authors repeatedly conclude that the 0.3 μg/kg dose of HSK21542 “significantly reduces pruritus and improves quality of life” and state that “the 0.3 μg/kg dose significantly reduced pruritus and improved QoL scores compared to the placebo group.” However, the data presented do not appear to support these claims. The leastsquares (LS) mean reduction in itch severity (WI-NRS) at 12 weeks (stated primary endpoint) was −3.47 in the 0.3 μg/kg group vs. −3.08 in the placebo group, a difference of - 0.39 with a 95% confidence interval (CI) of −1.42 to +0.64. This indicates no significant improvement over placebo. Indeed, the authors report this difference as non-significant (p > 0.4). This can also be observed in Figures 2a,b where the CIs of the placebo and the 0.3 μg/kg group overlap. I also note an error in the results: the CI for the 0.3 μg/kg vs. placebo is reported as “(1.42, 0.64)”, it should read −1.42 to +0.64. Considering the 0.3 μg/kg dose did not demonstrate a statistically superior anti-pruritic effect vs placebo, the claim that HSK21542 0.3 μg/kg “demonstrated superior efficacy” is not supported. Also, the claims of improved QoL seem overstated. The article reads that vs. placebo, the 0.3 μg/kg group showed “more significant improvements” and “greater improvements” in Skindex16 and 5-D Itch scores, but the differences have overlapping CIs, as reported in Table 1; Figure 4, indicating no statistical difference. In summary, the “superior efficacy” claim may be misleading. In addition, the claim of superior safety for HSK21542 is also unsubstantiated. The safety relative to placebo was comparable: the incidence of treatment-emergent adverse events in the 0.3 μg/kg group (76.7%) was similar to placebo (73.3%). Thus, asserting an overall superior safety is incorrect. The 0.3 μg/kg HSK21542 appears to have a safety/tolerability profile similar to placebo, which is reassuring, but not “superior”.

## High-dose (HSK21542 0.6 μg/kg) results

The findings for the higher 0.6 μg/kg dose which showed less itch relief vs. placebo is under-addressed. The group difference was +1.03 points vs. placebo, with a 95% CI of +0.01 to +2.07. Notably, this CI excludes zero, which implies a statistically significant worse result of the high dose vs. placebo. The authors do not explicitly acknowledge this but simply describe the 0.6 μg/kg effect as “less pronounced” than that of 0.3 μg/kg and “although various efficacy endpoints in the 0.6 μg/kg dose group showed improvement from baseline, no significant dose-response relationship was observed compared to the 0.3 μg/kg dose group.” These statements appear to understate the findings, as the data show a less favorable effect compared with placebo. I also note an omission in Table 2: the 5-D Itch score value is blank for the LS mean difference vs. placebo for the 0.6 μg/kg group, with only the CI provided, which may be a formatting error. In summary, the significant lower itch improvement at 0.6 μg/kg vs. placebo is an important result that warrants emphasis.

## Cross-trial comparisons

In the discussion section, the outcomes of this Phase II trial are compared with difelikefalin trials for CKD-aP. The authors report, that 62.1% of patients in the HSK21542 0.3 μg/kg group achieved ≥3-point WI-NRS improvement at 12 weeks, versus 49.1% and 53.4% in the Phase 3 KALM-1 and KALM-2 studies of difelikefalin, respectively. While these figures were correctly cited (Fishbane et al., 2020a; Topf et al., 2022), cross-trial comparisons are potentially misleading. The patient population and study conditions differed and no matching or statistical comparison was performed. Contextualization is important as directly comparing responder percentages in separate trials without adjustment may be misleading. The authors also draw parallels to the dose-response findings of difelikefalin’s Phase II trial. They state that the lack of a dose-response (0.6 vs. 0.3) in HSK21542 “mirrors the findings from the phase II study of difelikefalin, where the 0.5 μg/kg dose group exhibited better efficacy than the 1 μg/kg dose group.” This comparison may be imprecise as no statistical difference was observed between these groups, and the difelikefalin phase II trial showed a significant benefit or a trend towards a benefit of the drug vs. placebo at all doses tested (0.5 μg/kg being optimal) (Fishbane et al., 2020b). Given that the HSK21542 0.6 μg/kg dose group showed a statistically significant diminshed effect compared to placebo, drawing an analogy to difelikefalin may be misleading.

## Discussion

I recommend caution in interpreting the results of Pan et al. (2025) manuscript. The data do not demonstrate a significant benefit of HSK21542 0.3 μg/kg over placebo. I recommend that the authors and researchers ensure that statements in the abstract, discussion, and conclusion are fully supported by the results presented. Inadvertent mistakes, such as the missing number or minus symbol may be corrected to avoid confusion. Comparing results to other studies without direct comparative data should be made with caution. Accurate reporting of results with substantiated claims, will ultimately benefit the scientific community and patients by setting the stage for credible and reproducible findings in future trials. In summary, HSK21542 could be an interesting therapeutic candidate for CKD-aP, but the evidence from this Phase II study is not sufficient to prove superior efficacy and safety. We look forward to further studies that address these topics and clarify the role of HSK21542 in managing CKD-aP.
